# Supplementary material for: Impact of Body Composition Status on 90-Day Mortality in Cancer Patients with Septic Shock: Sex Differences in the Skeletal Muscle Index
Source: J Clin Med. 2019 Oct 2;8(10):1583. doi: 10.3390/jcm8101583 (PMC6832584; doi:10.3390/jcm8101583)
Supplement: Supplementary file 1 [file jcm-08-01583-s001.pdf]

Table S1. Demographic and clinical characteristics of study patients with and without muscle depletion.

Characteristics	Overall patients (n = 478)	Muscle depletion (n = 419)	No muscle depletion (n = 59)	P-value
Age, years	65.0 (58.0–72.0)	66.0 (58.0–73.0)	62.0 (56.0–67.0)	0.003
Male	297 (62.1%)	247 (58.9%)	50 (84.7%)	< 0.001
Comorbidities				
Hypertension	169 (35.4%)	147 (35.1%)	22 (37.3%)	0.740
Diabetes mellitus	124 (25.9%)	109 (26.0%)	15 (25.4%)	0.923
Type of solid cancer				0.401
Hepatobiliary	247 (51.7%)	212 (50.6%)	35 (59.3%)	
Gastrointestinal	91 (19.0%)	79 (18.9%)	12 (20.3%)	
Gynecologic	48 (10.0%)	46 (11.0%)	2 (3.4%)	
Lung	31 (6.5%)	28 (6.7%)	3 (5.1%)	
Others	61 (12.8%)	54 (12.9%)	7 (11.9%)	
Focus of infection				0.280
Hepatobiliary	243 (50.8%)	212 (50.6%)	31 (52.5%)	
Respiratory	63 (13.2%)	59 (14.1%)	4 (6.8%)	
Others	172 (36.0%)	148 (35.3%)	24 (40.7%)	
SOFA score	7.0 (5.0–10.0)	7.0 (5.0–10.0)	7.0 (5.0–9.0)	0.836
90-day mortality	208 (43.5%)	188 (44.9%)	20 (33.9%)	0.112
Body composition				
BMI, kg/m^2	22.0 (19.6–24.3)	21.8 (19.4–24.4)	22.8 (21.0–24.2)	0.029
SFA, cm^2	89.40 (53.71–144.18)	92.6 4(53.11–144.48)	87.40 (56.32–139.74)	0.820
VFA, cm^2	95.29 (56.96–134.44)	94.69 (57.61–138.64)	97.81 (50.97–124.92)	0.424
SMA, cm^2	89.64 (73.46–107.68)	84.84 (71.84–98.64)	125.75 (116.80–134.23)	< 0.001
SMI, cm^2/m^2	34.54 (7.58)	32.84 (6.36)	46.61 (3.44)	< 0.001
Skeletal muscle attenuation, HU	34.51 (6.94)	33.80 (6.80)	39.54 (5.71)	< 0.001
VFA/SFA ratio	1.07 (0.72–1.67)	1.07 (0.72–1.68)	1.02 (0.70–1.60)	0.483
Obesity	90 (18.8%)	81 (19.3%)	9 (15.3%)	0.453
Low skeletal muscle attenuation	202 (42.3%)	197 (47.0%)	5 (8.5%)	< 0.001

Data are presented as the mean (standard deviation), median (interquartile range), and number (percentage).

Abbreviations: BMI, body mass index; HU, Hounsfield units; SFA, subcutaneous fat area; SMA, skeletal muscle area; SMI, skeletal muscle area index; VFA, visceral fat area.

Table S2. Odds ratios for muscle depletion by logistic regression analysis in patients with cancer who developed septic shock.

	Univariable analysis		Multivariable analysis	
	**OR (95% CI)**	**P-value**	**OR (95% CI)**	**P-value**
Age, years	1.039 (1.013–1.065)	0.003	1.041 (1.012–1.070)	0.006
Male	0.258 (0.124–0.540)	< 0.001	0.277 (0.128–0.600)	0.001
Comorbidities				
Hypertension	0.909 (0.517–1.598)	0.740		
Diabetes mellitus	1.031 (0.552–1.928)	0.923		
Type of solid cancer				
Hepatobiliary	Reference			
Gastrointestinal	1.087 (0.537–2.199)	0.817		
Gynecologic	3.797 (0.882–16.353)	0.073		
Lung	1.541 (0.434–5.342)	0.495		
Others	1.274 (0.536–3.024)	0.584		
Focus of infection				
Hepatobiliary	Reference			
Respiratory	2.157 (0.732–6.354)	0.163		
Others	0.902 (0.509–1.599)	0.723		
SOFA score	1.017 (0.933–1.109)	0.706		
Low skeletal muscle attenuation	9.584 (3.758–24.438)	< 0.001	7.454 (2.891–19.219)	< 0.001

Abbreviations: CI, confidence interval; OR, odds ratio; SMI, skeletal muscle area index; SOFA, Sequential Organ Failure Assessment.

Table S3. Hazard ratios for 90-day mortality by Cox proportional hazards analysis in patients with cancer who developed septic shock.

	Univariable analysis		Multivariable analysis	
	**HR (95% CI)**	**P-value**	**HR (95% CI)**	**P-value**
Age, years	1.00 (0.99–1.01)	0.991		
Male	0.91 (0.69–1.20)	0.506		
Comorbidities				
Hypertension	1.03 (0.78–1.37)	0.838		
Diabetes mellitus	1.00 (0.73–1.36)	0.980		
Type of solid cancer				
Hepatobiliary	Reference			
Gastrointestinal	0.97 (0.67–1.39)	0.854		
Gynecologic	0.66 (0.38–1.13)	0.127		
Lung	1.51 (0.92–2.49)	0.105		
Others	1.07 (0.70–1.63)	0.771		
Focus of infection				
Hepatobiliary	Reference		Reference	
Respiratory	1.54 (1.05–2.24)	0.026	1.44 (0.98–2.10)	0.063
Others	0.86 (0.63–1.17)	0.340	0.86 (0.63–1.18)	0.346
SOFA score	1.05 (1.00–1.09)	0.046	1.04 (1.00–1.09)	0.052
SMI, cm2/m2	0.97 (0.95–0.99)	0.001	0.97 (0.95–0.99)	0.001
Low skeletal muscle attenuation	1.41 (1.08–1.86)	0.013		

Abbreviations: CI, confidence interval; HR, hazard ratio; SMI, skeletal muscle area index; SOFA, Sequential Organ Failure Assessment.
